# Dimerization of Firing Factors for Replication Origin Activation in Eukaryotes: A Crucial Process for Simultaneous Assembly of Bidirectional Replication Forks?

**DOI:** 10.3390/biology11060928

**Published:** 2022-06-17

**Authors:** Seiji Tanaka, Shiho Ogawa

**Affiliations:** School of Environmental Science and Engineering, Kochi University of Technology, Kami 782-8502, Japan; ogawa.shiho@kochi-tech.ac.jp

**Keywords:** DNA replication, origin firing, bidirectional replication, Sld3/Treslin/Ticrr, Sld7/MTBP

## Abstract

**Simple Summary:**

Chromosomal DNA must be faithfully duplicated and segregated into two daughter cells when cells divide. DNA synthesis initiates from specific regions known as the origins of replication. When it starts, a pair of the replication fork is established, and each replication fork moves away from replication origins. In each replication fork, replicative helicase unwinds DNA from a double to a single strand. This implies that two sets of active helicase are generated from each replication origin. To make this possible, two sets of replicative helicases are loaded onto replication origins as inactive dimers first. When S-phase specific cyclin-dependent kinases, S-CDKs, are activated, the inactive helicase is converted into the active helicase with the aid of other factors called firing factors. Although two sets of firing factors seem to be required to activate two sets of helicase, it is largely unknown whether two sets of firing factors function simultaneously to establish bidirectional replication forks in a coordinated way. We introduce our current understanding of firing factor dimerization and discuss its potential contribution to bidirectional replication fork formation in this review.

**Abstract:**

Controlling the activity of the heterohexameric Mcm2–7 replicative helicase is crucial for regulation of replication origin activity in eukaryotes. Because bidirectional replication forks are generated from every replication origin, when origins are licensed for replication in the first step of DNA replication, two inactive Mcm2–7 heterohexiameric complexes are loaded around double stranded DNA as a head-to-head double hexamer. The helicases are subsequently activated via a ‘firing’ reaction, in which the Mcm2–7 double hexamer is converted into two active helicase units, the CMG complex, by firing factors. Dimerization of firing factors may contribute to this process by allowing simultaneous activation of two sets of helicases and thus efficient assembly of bidirectional replication forks. An example of this is dimerization of the firing factor Sld3/Treslin/Ticrr via its binding partner, Sld7/MTBP. In organisms in which no Sld7 ortholog has been identified, such as the fission yeast *Schizosaccharomyces pombe*, Sld3 itself has a dimerization domain, and it has been suggested that this self-interaction is crucial for the firing reaction in this organism. Dimerization induces a conformational change in Sdl3 that appears to be critical for the firing reaction. Moreover, Mcm10 also seems to be regulated by self-interaction in yeasts. Although it is not yet clear to what extent dimerization of firing factors contributes to the firing reaction in eukaryotes, we discuss the possible roles of firing factor dimerization in simultaneous helicase activation.

## 1. An Overview of Origin Firing in Eukaryotes

In eukaryotes, chromosomal DNA replication is initiated from specific chromosomal regions known as the origins of replication. Activation of replication origins is achieved via two distinct reactions: licensing and firing, and these occur in this order. In the first reaction, licensing, the Mcm2–7 (mini chromosome maintenance) complex, which is the core component of the replicative helicase, is loaded onto replication origins as a head-to-head double hexamer, in which the N-terminal rings of each Mcm2–7 complex are adjoining, to form the prereplicative complex, pre-RC ([1], reviewed in [2,3]). In the second reaction, firing, which triggers initiation of DNA synthesis, the Mcm2–7 double hexamer is activated, and bidirectional replication forks including an active replicative helicase, the CMG (Cdc45 [cell division cycle]-Mcm2–7-GINS [5-1-2-3 in Japanese: go-ichi-nii-san]) complex, is formed [3,4,5,6]. Therefore, understanding how licensing and firing are regulated and occur, is crucial to understanding replication origin activation.

**Figure 1:**
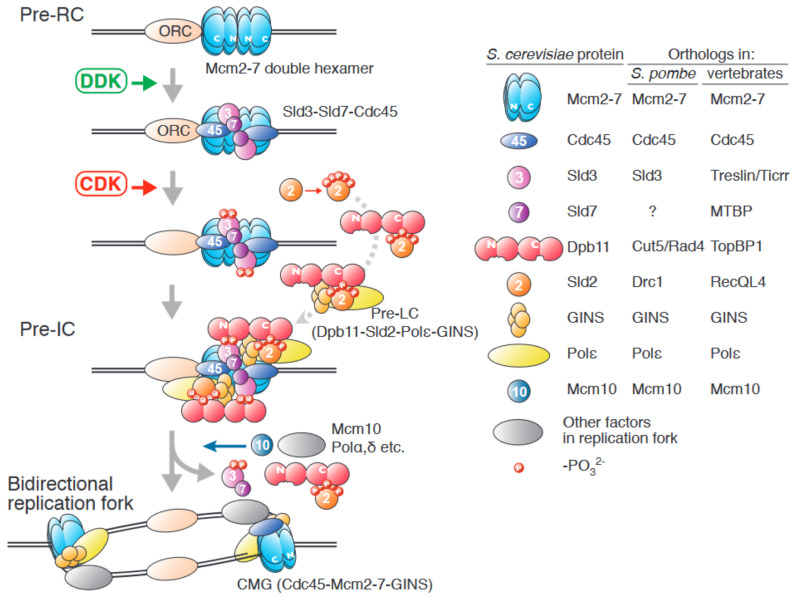
Schematic representation of the firing reaction in budding yeast. See text for details. The names of the orthologous proteins in *S. pombe* and vertebrates are shown in the table on the right.

Details of these reactions are best understood in the budding yeast *Saccharomyces cerevisiae*. In this review, we will focus on the firing reaction. For the licensing reaction, please refer recent publications [3,7,8,9]. An overview of the firing reaction is shown in Figure 1. This process can be divided into three steps, which are regulated by three factors: two conserved protein kinases, DDK (Dbf4-dependent kinase, also known as Cdc7–Dbf4 [dumbbell former] kinase) and CDK (cyclin-dependent kinase), and Mcm10.

In the first step, DDK phosphorylates the Mcm4 and Mcm6 subunits in the Mcm2–7 double hexamer, which promotes the interaction of Mcm2–7 and Sld3 (synthetically lethal with dpb11-1) [10,11,12,13,14]. The Mcm2–7 complex has two ring structures, the N-terminal and the C-terminal rings. In addition to these structured domains, Mcm2, Mcm4, and Mcm6 have the unstructured N-terminal tails at their N-terminal end that protrude from the ring structures in the double hexamer. DDK phosphorylates the N-terminal tail of Mcm4 and Mcm6 and the phosphorylated N-terminal tails work as Sld3-docking sites, although specific binding residues have not been defined [10,15]. Because Sld3 is already complexed with Cdc45 and Sld7 [16,17], this phosphorylation promotes Cdc45 loading onto the Mcm2–7 double hexamer.

Next, S-phase-specific CDK (S-CDK) phosphorylates Sld2 and Sld3 [18,19,20]. Phosphorylation of Sld2 promotes its interaction with Dpb11 (DNA polymerase B [II]) through its phospho-binding BRCT (BRCA1 C-terminus) domains [20,21], and further assembly of a complex containing DNA polymerase epsilon (Pol ε) and GINS, to form the preloading complex (pre-LC) [22]. The Dpb11 in the pre-LC also interacts with the phosphorylated Sld3 associated with the Mcm2–7 double hexamer, and thus, GINS in the pre-LC is also loaded onto the Mcm2–7 double hexamer. The resultant complex, assembled by S-CDK activation, is called the preinitiation complex (pre-IC) [23,24]. Dpb11 has four BRCT domains. The N-terminal BRCT pair of Dpb11 interacts with phosphorylated Sld3, and the C-terminal BRCT pair interacts with phosphorylated Sld2 [18,19,21]. In the pre-IC, the Mcm2–7 double hexamer is separated, and two sets of the CMG complex are assembled [14,24]. However, bidirectional replication forks are not formed at this point because the helicase activity of CMG in pre-IC is low.

Finally, Mcm10 activates the CMG helicase, bidirectional replication forks move away from the origins, and DNA is synthesized [14,25,26,27,28,29]. Sld2, Sld3, Sld7, and Dpb11 are all absent from the replication fork [5], suggesting that their roles are specific to the firing reaction (Figure 1).

The factors included in the pre-RC or the replication fork are well conserved in eukaryotes. In contrast, the regulatory factors of the firing reaction, such as Dpb11, Sld2, and Sld3, have diverged during evolution, although their orthologs are identified in model organisms. This may suggest details of the firing reaction are also slightly divergent in eukaryotes, although the reaction depends on CDK. For example, Sld2 has 11 putative CDK phosphorylation motifs. Among them, threonine 84 (T84) is essential for phosphor-dependent interaction with Dpb11, and phosphorylations at other sites enhance T84 phosphorylation [21]. In the fission yeast *S. pombe*, the Sld2 ortholog is Drc1 (DNA replication and checkpoint protein 1) [30,31]. Amino acid sequences of ScSld2 and SpDrc1 are similar (19.8% identical and 34.0% similar) and the critical CDK phosphorylation site is also conserved (threonine 114 [T114] in SpDrc1). T114 is the target of CDK, and phosphorylation of T114 is essential for DNA replication [30]. Therefore, the structure and the way for the regulation of the function are conserved between ScSld2 and SpDrc1. However, this seems not the case in the vertebrate Sld2 ortholog, RecQL4 (RecQ like helicase 4, also called RecQ4). Although RecQL4 is required for the initiation of DNA replication in *Xenopus* egg extracts, interacts with TopBP1 (DNA topoisomerase II binding protein 1), an ortholog of Dpb11, and shows a similarity to Sld2 in its N-terminus [32,33], RecQL4-TopBP1 interaction does not require CDK phosphorylation [32]. In contrast to the situation of Sld2, it seems that the regulation of the function of Sld3 is more conserved in eukaryotes. Details of this point will be described in the following section.

## 2. The Role of the Sld3/Treslin/Ticrr-Sld7/MTBP Complex in Origin Firing

As shown in Figure 1, bidirectional replication forks are generated from one replication origin. Therefore, at each origin of replication, two sets of every factor that functions in the firing reaction might be required. Moreover, additional mechanisms may exist to ensure that the two sets of firing factors function simultaneously to assemble two CMG helicases concurrently. Recent single-molecule analysis showed that bidirectional replication forks are formed per origin and progress away from origins at similar rates, which indicates the simultaneous assembly and activation of two sister-replisomes per origin [34]. If there is no such mechanism, firing factors are recruited to replication origins as two independent events. Such a firing mechanism may result in a low probability of both the assembly of two active sister-replisomes and the establishment of bidirectional forks from origins, and could cause growth defects by reducing replication fork numbers more than impeded firing.

In *S. cerevisiae*, the Sld3-containing complex includes Sld7 in addition to Cdc45 (hereafter referred to as 3-7-45) in vivo [17]. Sld7 possesses a self-interaction domain in its C-terminus, while the N-terminus of the protein interacts with Sld3 [35]. Therefore, two sets of the 3-7-45 complex can dimerize via the C-terminus of Sld7 and can thus be loaded onto Mcm2–7 double hexamer simultaneously. Sld7 is the only firing factor whose function is not essential for DNA replication, and hence cell growth, in budding yeast. Although *∆sld7* cells grow slowly, which might be caused partly by reduced Sld3 levels in the *∆sld7* cells, an increase in Sld3 protein in *∆sld7* cells is not sufficient for full recovery of cell growth [17]. Therefore, dimerization of 3-7-45 might be required for normal budding yeast cell growth. These indicate that the dimerization of 3-7-45 (at least by Sld7) is not essential for the firing, because Sld7 is not essential. This might suggest that the existence of a dimerization module other than Sld7 (see below) or concerted assembly of sister-replisomes is not essential. It is unknown whether origin firing with the uncoupled assembly of sister-replisomes and the generation of mono-directional forks are occurring in *∆sld7* cells. Therefore, it is intriguing to address this to understand the exact role of 3-7-45 dimerization.

Treslin (TopBP1-interacting, replication-stimulating protein)/Ticrr (TopBP1-interacting, checkpoint, and replication regulator) and MTBP (Mdm2 binding protein) are the vertebrate orthologs of Sld3 and Sld7, respectively [36,37,38,39,40]. These proteins, like their yeast orthologs, interact to form a complex [39]. MTBP contains two domains that show similarity with Sld7, S7M-N (Sld7-MTBP N-terminal), and S7M-C (Sld7-MTBP C-terminal) domains, and, as for Sld7, these domains interact with Treslin/Ticrr and MTBP, respectively [40,41]. In contrast to Sld7, MTBP is essential for cell growth [39]. Both S7M-N and S7M-C domains are important for DNA replication, suggesting that dimerization of the Treslin/Ticrr-MTBP complex is important for this process. Moreover, defects in DNA replication caused by deleting the S7M-C domain in MTBP can be rescued by addition of the GST (Glutathione S-transferase) dimerization domain to the MTBP protein lacking its C-terminus [40]. These suggest that simultaneous activation of the Mcm2–7 double hexamer by the Treslin/Ticrr-MTBP dimer is critical for cell growth in vertebrates (for details, please see the review by Zaffar et al. [42] in this special issue).

## 3. Dimerization of Sld3 in the Fission Yeast *Schizosaccharomyces pombe* Represents a Novel Mechanism for Regulation of Origin Firing

Core replisome components, such as the CMG helicase and DNA polymerases, are highly conserved in eukaryotes, and orthologs can easily be identified for a wide range of species using database homology search tools such as BLAST. By contrast, the amino acid sequences of firing-specific factors, such as Sld2, Sld3, Sld7, and Dpb11, are less well conserved. Thus, it is hard to find their orthologs in divergent organisms via simple homology search, although these proteins have some well-conserved stretches of amino acids, representing conserved domains [6]. The fission yeast *S. pombe* is a popular eukaryotic model system. It is often said that the budding yeast and fission yeast are as evolutionally divergent as yeasts and vertebrates; however, orthologs of Sld2, Sld3, and Dpb11 in fission yeast can easily be identified using BLAST searches. By contrast, to date, an Sld7 ortholog in *S. pombe*, the putative SpSld7, has not been identified. Attempts to identify SpSld7 by coimmunoprecipitation with SpSld3 have also been unsuccessful with our hands.

**Figure 2:**
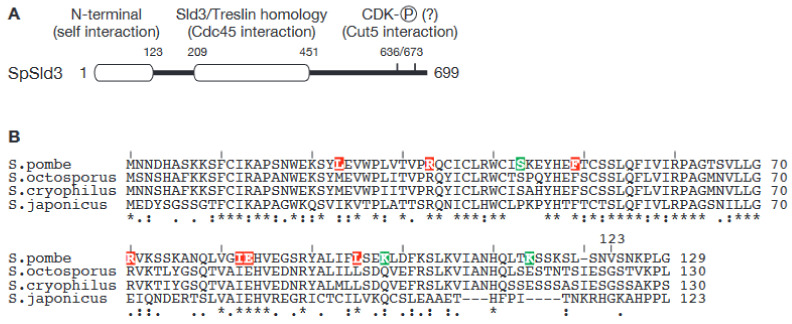
SpSld3 has *Schizosaccharomyces* specific N-terminal domain. (**A**). Schematic representation of the domain organization of SpSld3. (**B**). Multiple sequence alignment of N-terminal domain of Sld3 in *Schizosaccharomyces* species. Amino acids mutated in SpSld3N mutants that lack self-interaction are highlighted. Residues highlighted in red caused loss of interaction, while residues highlighted in green did not. “*” under the alignment indicates positions that have a single, fully conserved residue. “:” and “.” indicates conservation between groups of strongly and weakly similar properties, respectively. For more details, please visit the Clustal Omega web page (https://www.ebi.ac.uk/Tools/msa/clustalo/ (accessed on 8 May 2022).

Although it is unclear whether this protein exists in *S. pombe*, the *S. pombe* Sld3 (SpSld3) contains a self-interaction domain in addition to the conserved Sld3 motifs (Figure 2) [43]. Sld3 orthologs in budding yeast and vertebrates do not contain a dimerization domain, and thus, the ability of SpSld3 to self-interact is specific to SpSld3 and may bypass the requirement for Sld7.

The self-interaction domain of SpSld3 is found at the N-terminus, and the N-terminal 123 amino acids (SpSld3N) of the protein are sufficient for the interaction [43]. In SpSld3, the Sld3-Treslin domain (STD) is found in the middle of the protein, and crucial CDK phosphorylation sites that are conserved in Sld3 orthologs are found in its C-terminus. Neither of these regions overlap with the self-interaction domain, SpSld3N (Figure 2). Intriguingly, although the amino acid sequence of SpSld3N is highly conserved in all four species that belong to the *Schizosaccharomyces* genus (Figure 2B), no similar sequences were found in other species using database searches. Therefore, this self-interaction domain is likely to be very specific to the *Schizosaccharomyces* genus. Moreover, when eight point mutations that abolish the self-interaction of the SpSld3N fragment were isolated [43], in all cases, the mutations were in conserved residues (Figure 2B). This strongly suggests that the mechanism of self-interaction in SpSld3 is also conserved in all *Schizosaccharomyces* species.

Our recent analysis revealed that an SpSld3 mutant that lacks the self-interaction domain, SpSld3∆N, supports cell growth, although it shows mild replication defects, which might suggest that the self-interaction of SpSld3 is dispensable. However, full-length SpSld3 proteins harboring above-mentioned self-interaction-null point mutations showed severe growth defects. Furthermore, the interaction with Cut5 (cell untimely torn)/Rad4 (radiation sensitive) (hereafter referred to as Cut5, for simplicity), an ortholog of Dpb11 in *S. pombe*, is lost or diminished in these mutants. The SpSld3-Cut5 interaction is important for origin firing and is enhanced when SpSld3 is phosphorylated by CDK [30]. The crucial phosphorylation sites are found in the C-terminus of SpSld3 (Figure 2). This, therefore, suggests that self-interaction in the N-terminus of SpSld3 induces a conformational change that allows for the interaction of SpSld3 and Cut5 (Ogawa et al. manuscript in preparation). These results reveal a potential importance of self-interaction in the firing factor SpSld3 for the firing reaction and, in this case, for the function of SpSld3. This also suggests that it might contribute to simultaneous activation, instead.

**Figure 3:**
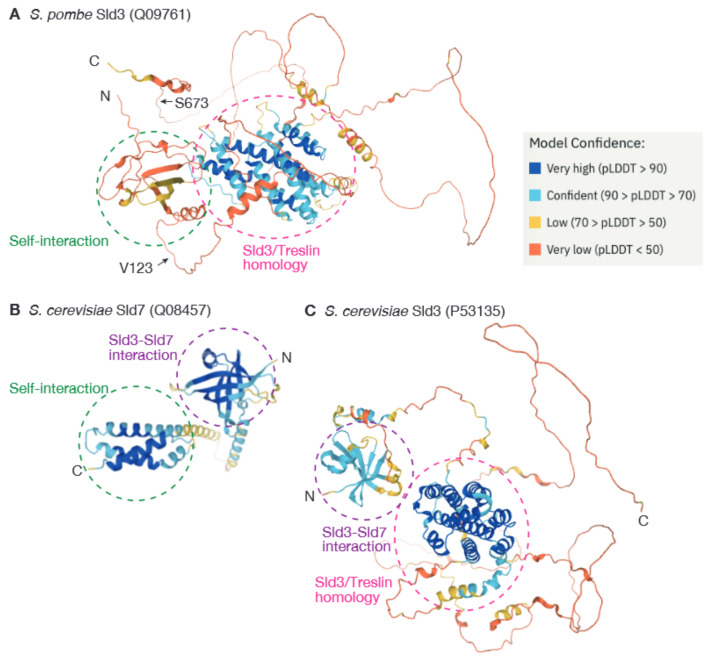
Structures of SpSld3, ScSld3, and ScSld7 predicted by AlphaFold2 (SpSld3: Q09761, ScSld3: P53135, and ScSld7: Q08457. Available online: https://alphafold.ebi.ac.uk (accessed on 8 May 2022)). (**A**–**C**). Self-interaction domains in SpSld3 and ScSld7 are indicated by green circles, Sld3/Treslin homology domains conserved in Sld3 orthologs are indicated by magenta circles, and the domain required for the interaction between ScSld3 and ScSld7 are indicated by purple circles.

A recently published SpSld3 structure, predicted by AlphaFold2, also supports this regulatory mechanism (Q09761. Available online: https://alphafold.ebi.ac.uk/entry/Q09761 (accessed on 8 May 2022)). Here, the SpSld3N domain is situated in close proximity to the C-terminus of the protein, which includes CDK phosphorylation sites, although the confidence values for the structural prediction of these regions are low (Figure 3A). Such proximity might be lost when SpSld3 dimerizes. Comparison of predicted structures of SpSld3N and the N-terminal portion of budding yeast Sld3, ScSld3, indicates that they are structurally very different. SpSld3N is also different from the dimerization domain of ScSld7 (Figure 3). The ability of SpSld3N to self-interact suggests that *Schizosaccharomyces* species do not require Sld7. The fact that no sequences similar to SpSld3N have so far been identified other than in *Schizosaccharomyces* species suggests that the N-terminal domain and regulatory mode of SpSld3 are evolutionarily unique.

## 4. Dimerization of Firing Factors May Ensure the Biological Robustness of the Firing Reaction

As shown in Figure 1, multiprotein complexes are recruited onto the Mcm2–7 double hexamer in a DDK- and CDK-dependent manner during the firing reaction. In budding yeast, the 3-7-45 complex is recruited in the DDK-dependent step [12,13]. Here, the association of Sld3 and Cdc45 with replication origins is mutually dependent in vivo, although such dependency cannot be observed in in vitro reconstitution systems [10,16,24]. In the next step, pre-IC assembly, which occurs in an CDK-dependent manner and assembles CMG, all pre-IC factors are essential in vivo [24]. As described above, the compositional difference between active and inactive helicase is just Cdc45 and GINS. However, other factors such as Dpb11, Sld2, Sld3, Sld7 and Pol ε are required for helicase activation. Although the exact reason why additional factors are required for the helicase activation is unknown, such factors might ensure the proper timing of helicase activation in the cell cycle by accepting the signal from CDK. In addition, the simultaneous requirement of firing factors in Cdc45 recruiting and the pre-IC assembly might reduce the possibility of premature firing of replication origins. In other words, such a regulatory mechanism increases the biological robustness of origin firing.

The eukaryotic genome contains many replication origins, and they fire gradually rather than all at once. Furthermore, each origin has its own timing for firing during S phase: some fire early and some fire late. This is called the temporal regulation/program of origin firing (reviewed in [44]). The association of 3-7-45 with the Mcm2–7 double hexamer regulated by DDK is central for this temporal program [45,46]. In budding yeast, the number of 3-7-45 complexes is limited, because their copy numbers are much less than the number of replication origins. Given that the loading of Sld3 and Cdc45 to origins is mutually dependent, and 3-7-45 seems to exist as a dimer, this limitation on 3-7-45 may contribute to ensuring correct execution of the temporal program.

It is still unclear to what extent the dimerization of Sld3/Treslin-Sld7-MTBP complex contributes to the biological robustness of the firing reaction by ensuring simultaneous activation of the Mcm2–7 double hexamer. As described above, although dimerization of 3-7-45 in budding yeast is not essential, *∆sld7* cells still show some phenotypes. In vertebrate cells, dimerization of Treslin-MTBP seems more important because disruption of dimerization causes a more severe phenotype [17,39,40]. However, no studies have addressed whether generation of a mono-directional fork rather than bidirectional forks from replication origins occurs in cells where dimerization of Tresilin-MTBP is disrupted or Sld7 is deleted. In fission yeast, dimerization of SpSld3 seems essential for DNA replication. Although this essentiality seems to come from the resultant conformational change of SpSld3, which is essential to promote the pre-IC assembly, it is still possible that dimerization itself contributes to ensuring simultaneous activation of the Mcm2–7 double hexamer.

It is also still unclear to what extent the dimerization of firing factors involved in subsequent firing steps contributes to origin firing. Mcm10 can self-interact via its zinc-finger motif, which seems to be evolutionarily conserved in both budding and fission yeast [47,48]. In both cases, mutations that disrupt the zinc-finger motif abolish Mcm10 self-interaction and cause cell lethality. Notably, the phenotype of the Mcm10^ZA^ mutant in *S. pombe*, in which three conserved amino acids in the zinc-finger motif are substituted with alanine, is intriguing. In yeast two-hybrid assays, wild-type SpMcm10 interacts with many replication factors included in the pre-IC, such as Mcm2, Mcm4, Mcm6, all four subunits of GINS, Cut5, Drc1 (Sld2 ortholog), subunits of Pol ε (Dpb2 and Cdc20), and Mcm10 itself. The Mcm10^ZA^ mutant retains all of these interactions with one exception, namely, Mcm10 self-interaction. Mcm10^ZA^ cells were unable to initiate DNA replication, and in the resultant Mcm10^ZA^-arrested cells, origin association of Mcm6, Sld3, and Mcm10 was observed, but origin association of Rpa2, a subunit of RPA (Replication protein A) single-strand binding protein, was not [47]. This suggests that in the Mcm10^ZA^-arrested cells, the pre-IC has been assembled, and Mcm10 is subsequently associated to the pre-IC, but robust DNA unwinding, which is essential for replication fork assembly, does not occur. These results might suggest that self-interaction of Mcm10 has a direct effect on DNA replication; however, Mcm10^ZA^ also has a reduced ability to bind ssDNA [47]. This may affect helicase activation in some way, although the exact role of Mcm10′s ssDNA-binding ability is not known. Therefore, further analyses are required to understand the effect of self-interaction of Mcm10 on the firing reaction.

Other budding yeast firing factors, such as Sld2, Dpb11, and Pol ε, also show self-interaction in the yeast two-hybrid assay (Tanaka et al., unpublished). However, because budding yeast is the host for the assay, it is impossible to distinguish whether such self-interactions are direct. Nonetheless, whether the interaction is direct or indirect, such self-interactions might suggest that the pre-LC itself dimerizes. Such a possibility is fascinating because it seems likely that at least two sets of pre-LC are required for activation of the Mcm2–7 double hexamer. The absence of dimerization of pre-LC might cause growth defects by impeding the simultaneous formation of two sister-replisomes, as in the case of *∆sld7* cells, in which the dimerization of 3-7-45 is absent and show some growth defects. Alternatively, self-interaction of these factors may induce conformational changes that regulate both assembly of the pre-LC itself and subsequent reactions, as for SpSld3. If this is correct, the dimerization process itself would increase biological robustness. Whatever the case is, as for spMcm10, further analysis is required to elucidate the biological role of such interactions. Recent single-molecule analysis on the firing reaction revealed that the DDK-regulated multiplicity of Cdc45-GINS binding onto Mcm2-7 double hexamer promotes assembly of the CMG [49]. Such observation might also support the importance of dimerization of firing factors.

As shown in Figure 1, DDK phosphorylates Mcm2–7. Intriguingly, DDK efficiently phosphorylates the Mcm2–7 double hexamer when loaded onto replication origins, but not free single Mcm2–7 hexamers [11]. Recent studies used cryogenic electron microscopy to explain this highly specific requirement of DDK [50,51]. These analyses demonstrated that within the Mcm2-7 double hexamer, DDK docks onto one MCM2–7 hexamer and phosphorylates the opposed hexamer. In the analysis, the Mcm2–7 double hexamer was observed bound to two DDKs. In these cases, the two DDKs are apart on the Mcm2–7 double hexamer, and no interaction was observed between the two DDKs [51]. Therefore, simultaneous phosphorylation of the Mcm2–7 double hexamer by dimerized DDK is unlikely. The absence of DDK dimerization suggests that the two Mcm2–7 complexes present in the double hexamer are not phosphorylated simultaneously. This may be because DDK is a protein kinase and, unlike firing factors, is not involved in providing structural support.

## 5. Concluding Remarks

In the firing reaction, the Mcm2–7 double hexamer in the pre-RC is activated and converted into two sets of CMG helicase, which promotes assembly of bidirectional replication forks. In this reaction, two Mcm2–7 complexes may be activated simultaneously by some regulated mechanism. Dimerization of firing factors may be the key to promoting the reaction itself, but such a mechanism would also increase biological robustness. Self-interaction of Sld7/MTBP may represent one such example. Several other firing factors show the ability to self-interact and thus may also contribute to simultaneous helicase activation. Further elucidation, including analyses of the self-interaction of these factors, is required to understand the mechanism for simultaneous helicase activation.

## Data Availability

Not applicable.
